# Self-Abortion

**Published:** 1882-01-20

**Authors:** Wm. H. Harrison


### SELF-ABORTION.

BY WM. H. HARRISON, M. D.

It is strange that some of the leading men of our profession are inclined to sneer at the possibility of a woman producing self-abortion. It is said that Dr. J. W. McLane, who succeeds Dr. T. Gaillard Thomas in the College of Physicians and Surgeons, delivered a lecture last winter to a jury, as expert, upon the impossibility of self-abortion. In this he seems to me to resemble that class of physicians who deny the existence of oxytocics, or the well-established physiological effects of some other most important drugs, doubting the experience of others simply because on their probable imperfect test of perhaps inferior articles these drugs failed to show their reputed power. He must see for himself the woman, the means, and the effect, and know personally from ocular demonstration that abortion has taken place as the effect of the means used by the woman only, before he will believe the possibility of self-abortion. As he has never seen a woman produce self-abortion, and fails to believe in the experience of others on this head, he assumes it to be an impossibility on a priori grounds.

I will give the particulars of a few cases which will illustrate the fact that a woman can not only produce self-abortion, but can do so scientifically.

In the fall of 1877 I saw Effie D., eighteen years of age. Four days before my first visit she miscarried, which was followed by puerperal peritonitis, for which latter trouble I was called to see her. Her elder sister gave me the following account of the case: Out of wedlock she had become pregnant. When three months gone in pregnancy she sought to produce abortion by the advice of an old woman, who told her to get a round stick, as large as she could introduce well into the vagina, round and smooth at one end, then introduce it and "job" the mouth of the womb with it as hard as she could "stand" it, and to repeat the procedure three or four times a day until the desired effect was produced. After the third day's "jobbing" she was taken with labor-pains, and within eight or ten hours abortion was completed.

The following case shows the application of scientific means to create self-abortion:

In the spring of 1879 I was summoned by a servant girl in great haste to see Mrs. G., the wife of a railroad man, whom she said was in a dying condition. I found the patient unconscious, and while examining for a cause of her condition I noticed that she was having something very much like labor-pains. I inquired of the servant if the patient was in the "family-way," but she said she didn't know, and all I could learn from the girl was that while in an adjoining room she heard the patient scream, and when she got to her she was in the unconscious state in which I fouud her. I was soon convinced, however, that my patient was having labor-pains, and at once made a digital examination; and you may judge of my surprise when I found what subsequently proved to be a small rounded piece of whalebone, ten or twelve inches in length, protruding from the vagina. Further examination revealed the smaller end lying loosely in the mouth of the womb, which was considerably dilated. I removed the whalebone and in ten or fifteen minutes the womb expelled a fetus of perhaps three and a half or four months. My patient soon rallied and became very talkative and free to explain, after the servant girl, who was the only other person in the house, left the room.

She explained as follows: Three years previously, while residing in Chicago, she went to full term in her first pregnancy, and the child had to be taken from her in pieces. In a year she again became pregnant, when upon consulting her physician, the same who attended her before, he advised the propriety of an early abortion, to which she and her husband readily agreed. She was then about three months advanced, and the operation was performed by the physician introducing a long flexible instrument (perhaps a gumelastic bougie) into the womb and leaving it there; she lying in bed from the time of its introduction until she aborted. Again becoming pregnant, having left Chicago, being about three months advanced, and fully determined on abortion she decided to undertake the operation herself. I will say just here that she was a woman of unusual intelligence, and knew more about herself than most women do about themselves. So she procured the piece of whalebone and prepared it very nicely; she then went to bed, and with the forefinger of the left hand found the mouth of the womb, and with the right hand succeeded after a while in introducing her improvised probe. In about three hours she began to feel some pain, which gradually grew worse, and the last she remembered until "all was over" was a very severe pain, which seemed to extend from her womb to her head.

A very common practice among the negro wenches of the South inducing self-abortion is by jumping from high places, such as a fence or a gatepost to the hard ground. I believe that a very large majority of self-abortions as well as all other superinduced abortions are produced by the use of oxytocics, principally cottonroot and ergot. I think I am warranted in estimating the proportion of self-abortions as at least one-third of all that are superinduced.—[Louisville Medical News.

